# When Average is Over: Small N but Many Trials

**DOI:** 10.5334/joc.168

**Published:** 2021-08-27

**Authors:** Alex O. Holcombe

**Affiliations:** 1 School of Psychology, the University of Sydney, AU

**Keywords:** Attention, Visual perception, Mathematical modelling

## Abstract

Rouder & Haaf (2021) provide a valuable recipe for testing whether there are qualitative differences. This should hasten the day when psychologists routine consider individual participant data, rather than just the average of the participants’ data. Work remains to be done, however, on how to approach the issue of individual differences with the small-N, many-trials tradition that dates back to the beginning of experimental psychology and continues today in some areas, particularly cognitive modelling and perception.

An experimental psychologist decides to follow up on a preliminary report (Alighieri, 1320) suggesting that the conditions in Hell are inhumane. The psychologist uses an opportunity sample drawn from the 4th ring of the ninth circle of Hell and the first ring of the 7th circle of Hell, not realizing that this means that half his souls are frozen solid while the other half are in a river of boiling fire. The psychologist calculates the mean temperature of his participants and concludes that souls in Hell are housed at approximately 21° Celsius, a quite comfortable clime. The researcher does note his sample’s large standard deviation and the associated large error bars on his point estimate, so he calls for more research with a larger sample, as the average temperature in Hell can then be more precisely estimated. The line of research is continued by other psychologists, who begin by conducting a power analysis to arrive at the sample size they’d need to reject the null hypothesis that the average temperature in hell is 21°C.

The above parable highlights the sometimes-diabolical nature of customary statistical practices in psychology. The limitations of some of these practices can be overcome by using the statistical recipe of Rouder & Haaf (2021) for testing whether a dataset contains evidence of qualitative individual differences. This would have been very illuminating in the situation of the above parable, where the sample of participants reflected two populations that are qualitatively different: half hot and half cold.

The possibility of qualitative differences in psychology data sets continues to be systematically neglected by many researchers. This is sometimes sensible for certain between-participant methods designs, but unfortunately this habit is also now prevalent in areas that use the within-participant, many-trials method that dominated in psychology’s early history.

Early researchers such as Ebbinghaus, Weber, and Fechner discovered and documented their phenomena by collecting large amounts of data on just one participant – themselves. Their findings have stood the test of time. And these early efforts to collect lots of data from each participant were not confined to self-experimentation. When experimental psychology labs were founded in the 1890s, “the students worked with each other and even with the professor as observers” (Boring, 1954) rather than recruiting large numbers of people.

## Fields of small N, but many trials

Still today, many researchers who study perception – and some in a few other areas such as cognitive modelling – make the majority of their discoveries by studying themselves. For those researchers, additional participants are sometimes thought of as necessary only to convince the world of what the researcher already knows. I hasten to say that I do not know of any data documenting this attitude, so you may consider this a discovery I made about myself that I also believe is true of others (irony alert).

In one version of the small-N tradition, a researcher will first plot the data she collected on herself. Statistical inference is done on that data alone, either with an explicit test or “by eye”, assisted by inclusion of confidence intervals in the plot. The researcher then proceeds to get data from others, plotting successive participants’ data separately and running any statistical tests on each one. Each additional participant, then, is essentially a replication experiment (see Smith & Little, 2018 for discussion). I will refer to this as the “repeated N-of-1” framework.

Many psychology journal editors likely are not aware of the repeated N-of-1 framework and, influenced by psychology’s replication crisis, expect researchers to use the large numbers of participants one would need to show statistical significance in a between-participant analysis. That is an issue for another time.

Within the repeated N-of-1 framework, data from an individual participant constitutes a full experiment, with the additional participants serving as replications. In this situation, the problem of assessing qualitative individual differences is different than the way that Rouder & Haaf (2021) frame it. One could use hierarchical modelling a la Rouder & Haaf to test for between-participant differences, but first I would like to lay out the problem for the traditional perception approach of each participant analyzed separately.

As a repeated N-of-1 experiment proceeds, the test statistic applied to each participant may indicate that for some participants, the effect is significantly greater than zero, whereas for other participants, the effect is significantly less than zero. This could constitute good evidence for qualitative differences.

A problem, however, is that the decision threshold applied to each participant (such as *p* < .05 or a Bayes Factor greater than some criterion) will occasionally result in an error if the number of trials is limited enough that the within-participant aggregate has non-negligible variance. The large number of trials available for each participant should typically make the confidence interval around the estimate of the effect for each participant rather small, but it may not be negligible.

Let’s say that a *p*-value is the statistic used for inference in each participant. As the associated statistical test is applied to each participant individually, each has a certain probability of being in error (i.e., a false positive or a false negative). In other words, due to multiple comparisons across multiple participants, the false positive and false negative error rates are inflated above their nominal values. That is, as the number of participants increases, the probability increases that at least one statistical test will suggest a qualitative individual difference where there is none.

In the mainstream large-N approach to psychology research, the inflation of type 1 error rates is sometimes addressed by adjusting alpha (Ryan, 1959). I have never seen this done, however, in repeated N-of-1 studies. The status quo in perception research is that no statistical or other formal approach is typically applied to the issue of whether qualitative individual differences are present. The individual differences suggested by the data in perception experiments (quantitative or qualitative) are frequently passed over by the authors without any comment. I suspect that many researchers are motivated by a desire to sweep the apparent differences under the rug. When differences *are* remarked upon, they are often described vaguely, sometimes as a difference in “observer strategy”, and in subsequent papers, such findings are rarely referred to, even when they are relevant. The study of the flash-lag effect (Nijhawan, 1994; Metzger, 1932) provides one example.

The flash-lag effect is often explained by the proposition that the visual system extrapolates the position of moving objects to compensate for neural latencies (Nijhawan, 1994; 2008). The existence of participants who do not show the effect or show it in the opposite direction would not sit well with this extrapolation theory, because the theory seems to conceive of extrapolation as a basic function that all typically-developing humans should exhibit to some degree. If qualitative individual differences exist, they would sit more easily with theories of the flash-lag effect based on attention or asynchronous feature binding (e.g., Shiori et al., 2010; Murai & Murakami, 2016) rather than extrapolation.

Most papers on the flash-lag effect report data from very few participants, usually fewer than eight, although this may be changing in this era when many journal editors habitually demand larger Ns.

In a paper testing just four participants, my collaborators and I found that one participant showed the effect in the opposite direction of that usually found. We also noted that the degree of *within-*participant *variability* (SD ≈ 70 ms) of the effect was more similar between participants than was the magnitude (and possibly direction) of the effect (Linares, Holcombe, & White, 2009). That is, not only is it possible that qualitative individual differences exist, but also the within-participant variability of the effect may be more consistent than the magnitude of the effect. Because temporal mis-binding and attentional theories predict a certain degree of within-participant variability but the extrapolation theory does not, confirmation of qualitative differences and relatively-consistent within-participant variability would support the former theories. Unfortunately, however, this has not been considered even in papers that have enough data to test for it.

Two such articles used 24 and 25 participants (Chota & vanRullen, 2019; Morrow & Samaha, 2021). In one of these studies, the data from 4 (out of 25) participants was in the opposite direction of the usual effect, and in the other paper 3 participants (out of 24) showed such a result. If the seven contrary participants are a result of true qualitative individual differences rather than trial noise, they constitute a problem for the extrapolation theory. Unfortunately, neither paper commented on the possibility that they had uncovered true individual differences. Their Ns may be large enough, however, that the hierarchical modeling approach of Rouder & Haaf could be used on their data to assess whether the individual differences observed include qualitative differences.

With the provision of easy-to-use R code by Rouder and Haaf (2021), we researchers now have less excuse for ignoring the possibility of individual differences. I hope that the work of Rouder & Haaf will usher in a new era, one in which we can say honestly that “average is over!”.
